# Pediatric rigid bronchoscopy and foreign body removal during the COVID-19 pandemic: case report

**DOI:** 10.1186/s40463-020-00464-z

**Published:** 2020-09-14

**Authors:** Darren Jonathan Leitao, Jodi L. P. Jones

**Affiliations:** Department of Otolaryngology-Head and Neck Surgery, Rady College of Medicine, University of Manitoba, Room GB421-820 Sherbrook Street, Winnipeg, MB R3A 1R9 Canada

**Keywords:** COVID-19, SARS-CoV-2, Bronchoscopy, Foreign body aspiration”, “Case report”

## Abstract

We present the case of an eight year old boy who presented with foreign body aspiration during the COVID-19 pandemic. The patient was taken the operating room for rigid bronchoscopy and foreign body removal. The details of the operation, steps taken for protection of health care workers, and lessons learned are discussed. Bronchoscopy was performed using N95 respirators and Stryker Flyte Hood garments, combined with a streamlined instrument set-up. Simulation in advance of these cases improves communication and operative planning. Surgeons should have equipment to retrieve foreign bodies from the oropharynx available. Techniques that reduce surgical time and thus exposure risk should be considered.

## Background

Foreign body aspiration is a potentially critical airway emergency in children. Our current state of the art is endoscopic foreign body retrieval using rigid bronchoscopes and optical forceps for optimal imaging and surgical removal. These techniques are performed in children that are spontaneously ventilating under general anesthetic, exposing the surgical team to patient generated aerosols. The 2019 novel coronavirus disease (COVID-19) pandemic has dramatically changed our comfort with these procedures. Aerosol-generating medical procedures (AGMPs) pose significant risks of transmission of the sars-coV-2 virus to healthcare workers (HCWs) [1]. There is significant concern among otolaryngologists regarding their risk of occupational exposure during AGMP’s [2], especially since the first physician death from COVID-19 globally was an otolaryngologist in Wuhan, China [3].

Bronchoscopy with foreign body removal is an AGMP that must often occur in emergent, life-threatening situations, precluding our ability to obtain pre-operative confirmation of negative COVID-19 status. The use of administrative and environmental protocols and personal protective equipment (PPE) by all operating room staff are therefore critical to ensuring the safety of HCW’s in this operating room setting. Several protocols for pediatric airway management have been developed worldwide, as well as unique modifications to standard practice, to reduce exposure to aerosols intra-operatively [4, 5]. These protocols have introduced and incorporated unique surgical draping over the patient to trap aerosols during bronchoscopy, minimized equipment and personnel in operating environments, and outlined the PPE requirements for staff. This paper will highlight our experience of a case of bronchoscopy for foreign body removal under emergent conditions, and our operating room processes developed to manage these cases during the COVID-19 pandemic. We discuss some areas for improvement and consideration.

## Case presentation

**Fig. 1:**
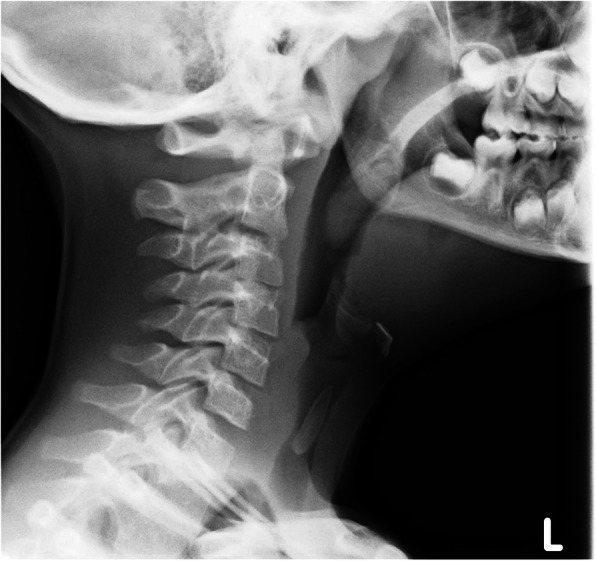
Lateral neck x-ray demonstrating foreign body lodged in upper trachea

Patient X is an eight-year-old boy who presented to the Children’s Hospital at HSC-Winnipeg in early April 2020 with foreign body aspiration. He had a week-long history of cough and respiratory symptoms that began after eating sunflower seeds. The patient had no other symptoms of COVID-19 disease at the time (no fever, sore throat, rhinorrhea) but did have paroxysmal coughing and shortness of breath. Patient X’s past medical and surgical history were unremarkable, and the patient had no known travel, contacts, or risk factors for COVID-19. The patient had been transferred from a community hospital, where a lateral neck X-ray and chest X-ray had revealed a large opaque foreign body consistent with a seed located in the upper trachea (Fig. 1). The patient was noted to have increased work of breathing, and biphasic stridor was noted throughout the chest. The Pediatric Otolaryngology service was consulted, and plans were made for emergency bronchoscopy and foreign body removal.

**Fig. 2:**
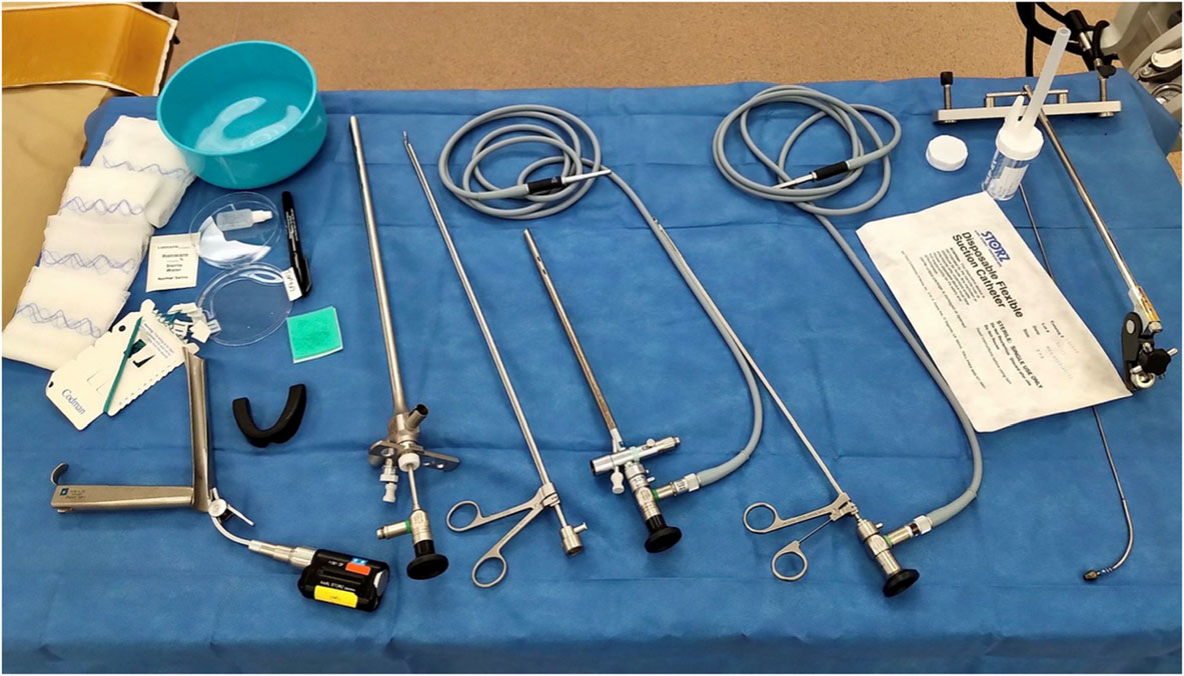
Airway instrumentation table set-up. Note dual telescope-light cord setup, preloaded into bronchoscope and optical forceps

The surgical team consisted of two pediatric anesthesiologists, a senior pediatric otolaryngologist, and two operating room nurses. The case was deemed emergent, and therefore not appropriate to wait for pre-operative COVID-19 testing and confirmation of negative status. Point of care testing was not available at our institution. The operation itself was conducted in a negative pressure theatre identified to require 30 min of time for full air exchange. All extraneous equipment was removed from the room. Appropriate signage was displayed on the doors to the theatre that identified this case as a possible COVID-19 case. Doffing locations within and outside the operating theatre were identified. All operating room personnel wore N95 masks, Stryker Flyte Hoods, long sleeve surgical gowns, and extended cuff gloves. Bronchoscopic equipment and appropriate optical forceps were selected prior to the patient entering the theatre. We used a two “camera-telescope-light cord” setup, one for the diagnostic bronchoscopy, and the other loaded into the optical forceps (Fig. 2). Several bronchoscopes and laryngoscopes were selected and available. Instrumentation was organized to be passed smoothly to the surgeon’s hands, while light cords were directed towards the back of the table for easy access and plugging into the video tower light sources. A flexible suction and suction trap were available for bronchial secretion collection.

**Fig. 3:**
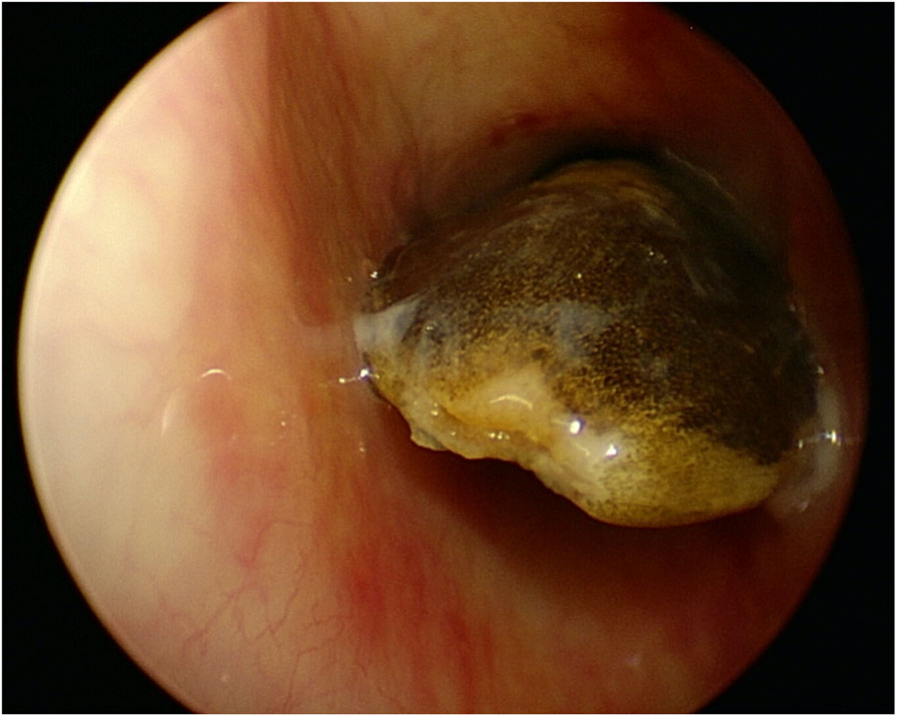
Endoscopic view of foreign body (sunflower seed) lodged in airway, just below cricoid cartilage

The patient was brought to the operating theatre wearing a standard surgical mask for droplet precautions. Anesthesia was performed with an intravenous technique, maintaining spontaneous ventilation. The vocal cords were sprayed with local anesthetic by the anesthesiologist using a video laryngoscope and a droplet applicator. Once the appropriate plane of anesthesia was achieved, the patient’s surgical mask was removed, laryngoscopy performed. The epiglottis and vocal cords were unremarkable in appearance. A size 5 × 30 cm rigid bronchoscope (Storz) was advanced into the airway, and a large sunflower seed was identified, lodged at the inferior aspect of the cricoid cartilage (Fig. 3). The seed was so large it could only transit between the inferior border of the cricoid cartilage and the carina. The optical forceps could only grasp the seed at its’ front aspect as the seed was so large; it could not be withdrawn completely into the bronchoscope. The seed, forceps, and bronchoscope were all removed from the trachea and the larynx together; however, once past the glottis the seed was inadvertently dropped into the oropharynx.

We attempted to expose the oropharynx and foreign body with the Parsons laryngoscope, however its straight design and narrow aperture made it difficult to get adequate exposure. We intubated the patient in order to ensure the foreign body did not return to the airway. The standard anesthetic laryngoscope with Macintosh blade proved to provide sufficient exposure to visualize the foreign body and remove it with the optical forceps. Removing the foreign body from the oropharynx with the 36 cm optical forceps was successful, but cumbersome to perform. The ideal tool to remove the foreign body from the oropharynx would have been a Magill forceps; this was not present in the room for the case. Repeat bronchoscopy assessment revealed no secondary foreign bodies. We aspirated secretions from the right upper lobe bronchus with a size 7 French flexible catheter and sent for viral testing. Suction was clamped before and after aspiration. The patient’s standard surgical mask was once again replaced over the mouth and nose.

After waiting 30 min for air exchange to be completed, the surgical team proceeded with doffing their personal protective equipment. We did this one at a time, beginning with the most experienced nurses in the room. We doffed our gowns and gloves in the operating room, and then removed our Stryker Flyte Hoods and N95 masks outside the theatre in a designated doffing area. An additional nurse was available outside the operating room to assist with the process and to ensure doffing was done correctly. Each person required approximately 5 min to correctly doff their personal protective equipment for a total of 25 min of time from beginning to end of doffing process for the surgical team. Once doffing was complete, the patient was transferred to the recovery room where staff with appropriate personal protective equipment were present to take over care. The patient was subsequently transferred home in stable condition later that same day. The patient’s bronchial aspirate returned a “negative” result 3 days later.

## Discussion and conclusions

Despite the COVID-19 pandemic, pediatric airway emergencies will still occur, and will require pediatric surgeons to provide operative intervention emergently, without confirmation of a child’s COVID status. This case highlights steps we took to perform critical pediatric airway surgery during the COVID-19 pandemic. There are several learning points that we wish to share with our colleagues, which are listed here.

First, we recommend performing all cases as if they are a sterile procedure. This will allow the surgical teams to learn consistent donning and doffing routines for PPE in a consistent manner across all manner of cases. We feel that the less variation there is in these donning and doffing procedures, the less chance there is for accidental contamination during the process.

Second, simulation and preparation are important. Simulation enables the team to state their concerns in an open forum, discuss everyone’s perceptions of safety and comfort, and to practice any techniques that are new modifications during the pandemic. A fortunate occurrence was that the operating room staff had just completed bronchoscopy simulation sessions the day prior to this case. These sessions allowed staff to express their anxiety and concerns about issues such as anesthetic technique, equipment and personnel needs, and PPE needs. We tried to implement several new draping techniques that have been discussed nationally and internationally [4, 5] and found we could not create a level of comfort with these modifications for the entire surgical team. This process ultimately led to a cohesive, collaborative plan for emergency airway surgery for our team.

**Fig. 4:**
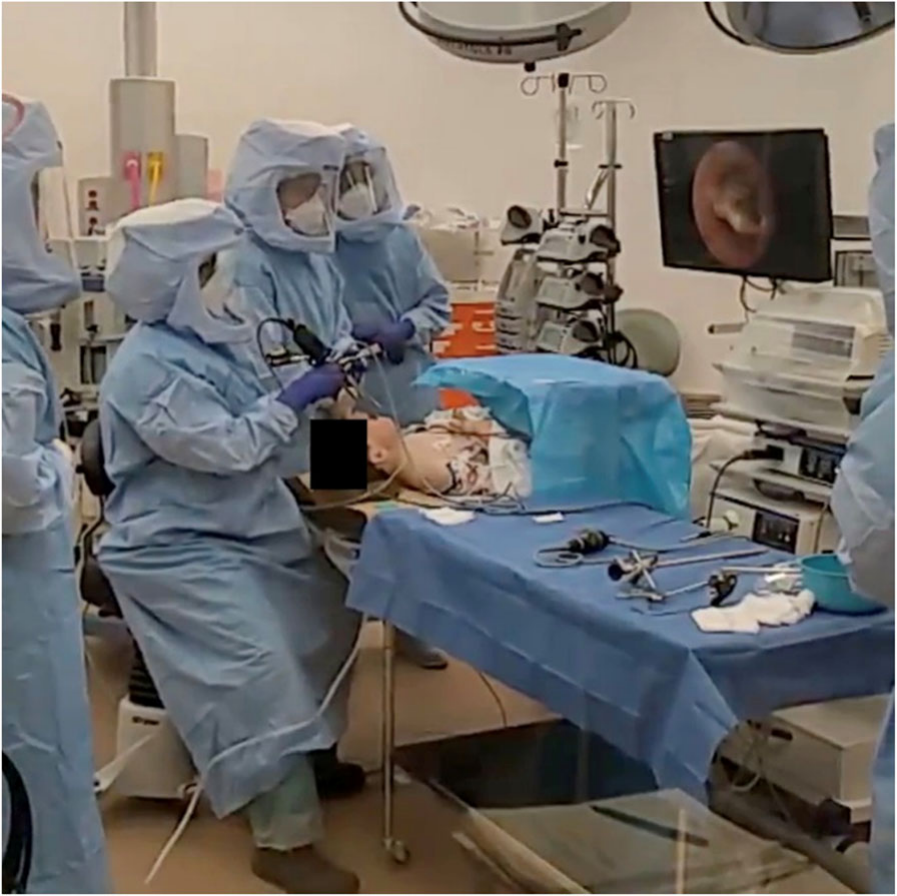
Surgical team performing bronchoscopy in PPE. Note PPE used and positioning of equipment within the room

Third, there is much debate regarding the availability, necessity and benefit of powered air-purifying respirators (PAPR) for AGMPs [6]. We did not use PAPR’s. For PAPR usage to be effective and safe, a broad-based training program for all operating room nurses, anesthesiologists, and surgeons involved in AGMPs would be required. In our province, 42 PAPRs exist, however there is only one clinician (an anesthesiologist) who is trained in the proper donning and doffing of such equipment. Significant resources would be required to adequately train a representative cohort of anesthesiologists, surgeons and nursing staff in order to safely implement PAPRs for routine use with airway emergencies. In our current system, this cannot yet be established. We used the N95 mask respirators (3 M) for aerosol protection, and Stryker Flyte Hood combined with level 4 surgical gown for enhanced droplet precautions (Fig. 4). The Flyte Hood provides Association for the Advancement of Medical Instrumentation (AAMI) level 4 (i.e. “highest”) droplet protection to the front face, neck, and shoulder regions, the areas with direct exposure to the patient’s airway. Although not a respirator, studies have shown that the Flyte Hood reduces exposure to environmental particles by a factor of 4.8, with the top of hood filtering 98% of particles greater than 0.1 microns [7]. We found the Stryker Flyte Hood to be lightweight and easy to wear and provided full face and neck protection. Our team felt that wearing the Stryker garment was a good surrogate to using extra draping over the patient, with the added benefit of not interfering with manipulation of the operative equipment. The Stryker Flyte Hood was familiar to our surgical team and was easy to incorporate into our existing routines.

There is understandably concern about whether the N95 FFR is protective enough. Patel et al. comments that otolaryngologists in China have contracted the virus despite use of the N95 mask, and that adequate control was not achieved until PAPR’s were utilized [2]. There are several reasons why the N95 mask may not have protected against exposure. Filter penetration by virus is one reason for concern, but there are more likely contributors such as poor fit and face seal and improper maintenance [8]. According to 3 M research, even though the N95 mask filters 95% of particles less than 0.3 μm, its filter efficiency is an inverted bell curve, with increasing filter efficiencies at particle sizes less than 0.04 μm and greater than 0.1 microns [8]. The SARS-CoV-2 virus is measured at 0.12 μm and average cough bioaerosol droplet sizes are measured larger than this, with 97% of droplets between 0.12 and 1 μm size [9]. It was felt that the combination of the N95 FFR with the Stryker Flyte hood provided our team with the best balance of aerosol and droplet protection, while providing full face and neck protection in a garment that was familiar to operating room staff with respect to donning and doffing. Additional aerosol protection can be achieved with higher level respirators (N99, N100).

Fourth, we feel that for emergent airway cases, keeping anesthetic and surgical processes as close to usual processes will reduce operative risks. As processes and protocols become more complex in our quest for the “perfect, 100%” protection, each change creates an extra layer of training, opportunity for error, and variability in outcome. It is known from previous reports that duration of exposure time to patients with SARS was a risk factor for HCW’s contracting the illness [10]. If an operative process can be performed as close to usual process as possible, then the procedures can be done as efficiently as possible, thereby reducing exposure time and risk. Some airway protocols for COVID-19 advocate for rapid sequence induction (RSI) and endotracheal tube (ETT) insertion for AGMP’s to avoid aerosol generation [11]. There are risks with this approach for patients presenting with upper airway obstruction. If we had paralyzed the patient, taken over ventilation, and intubated, there were potential for airway risks: 1) lodging of foreign body into endotracheal tube; 2) pushing the ETT deeper may have flipped the seed horizontally to obstruct both bronchi; 3) mechanical ventilation could have provided higher airway pressures to push the foreign body into right mainstem bronchus, thereby creating single lung ventilation and potentially worsening respiratory status. These issues were minimized by maintaining spontaneous ventilation and minimizing airway instrumentation to only the surgeon via bronchoscopy.

There has been discussion of using clear drapes over surgical field to reduce/eliminate aerosol generation [5]. We considered whether additional draping over the patient would provide additional protection against aerosolization during rigid bronchoscopy. When attempted in simulation we could not get it to work well enough to make the team feel safe. There are drawbacks to additional draping: set-up time, equipment manipulation, and risk of contamination. We practiced usage of a clear drape over the surgical field in simulation but were unsuccessful in creating a system that created a reliable, sealed working area. We worried about the additional time it might take to operate under the extra draping and encountered challenges in passing instrumentation during simulation. For the surgeon, the large movements of the right arm as it manipulates the optical forceps proved difficult to maintain the drapes in position. As a result, our team felt that enhanced PPE for staff was superior to additional environmental controls, and that our standard anesthetic and surgical technique would allow for the fastest procedure with the least exposure time for HCWs. As new techniques evolve and with more opportunities to practice in simulation, the use of additional draping techniques may allow for preservation of critical PPE while still maintaining safety for operating room personnel.

Lastly, it is important to ensure that all potential equipment needs are considered and available in the operating theatre prior to the start of the case. In our case, we selected several different sizes of bronchoscopes, and had pre-assembled bronchoscopes and optical forceps with telescopes, light cords, and camera heads to minimize equipment manipulation during the case. During the foreign body removal, the seed was dropped in the pharynx, just above the vocal cords. The process of improving safety with administrative controls resulted in the removal of all non-essential anesthetic equipment out of the room; among this equipment was the Magill forceps, a tool commonly used by the surgeon (and anesthesiologist) to remove items from the oropharynx. This meant that the Magill forceps were not available in the theatre when called for. This was a scenario that, while common, was not anticipated. Dropping the foreign body outside the larynx was not considered in our simulation, and therefore we were not prepared for this. We would suggest surgeons keep the Magill forceps available for these procedures.

In summary, pediatric rigid bronchoscopy with foreign body removal will be an uncommon but necessary procedure for pediatric otolaryngologists to perform during the COVID-19 pandemic. These patients will present in airway distress with a variety of respiratory symptoms that may mimic COVID-19 symptoms. Airway emergency simulation exercises are an invaluable resource to identify critical areas for improvement, especially when considering the dramatic changes to the operating room environment and surgical techniques during the COVID-19 pandemic. It is important for surgical teams and pediatric institutions to have discussed and developed a process for dealing with this problem, within the framework of each institution’s hospital policies, resources, available PPE, and simulation capabilities. We suggest that the combination of N95 mask or better, a hooded face-shield/barrier garment, and the simplest anesthetic and surgical technique afford the best way to limit exposure risks to operating room staff.

## Data Availability

Data sharing not applicable to this article as no datasets were generated or analyzed during the current study.

## Abbreviations

COVID-19: 2019 novel coronavirus disease
AGMP: Aersol-generating medical procedure
HCW: Health care worker
PPE: Personal protective equipment
PAPR: Powered air-purifying respirator
AAMI: Association for the Advancement of Medical Instrumentation
RSI: Rapid sequence induction
ETT: Endotracheal tube
